# Spatial connectivity of reef manta rays across the Raja Ampat archipelago, Indonesia

**DOI:** 10.1098/rsos.230895

**Published:** 2024-04-10

**Authors:** Edy Setyawan, Mark V. Erdmann, Ronald Mambrasar, Orgenes Ambafen, Abdi W. Hasan, Muhamad Izuan, Imanuel Mofu, Mochamad I. H. Putra, Abraham B. Sianipar, Rochelle Constantine, Ben C. Stevenson, Fabrice R. A. Jaine

**Affiliations:** ^1^ Institute of Marine Science, University of Auckland, Auckland 1010, New Zealand; ^2^ Conservation International Aotearoa, University of Auckland, Auckland 1010, New Zealand; ^3^ West Papua Program, Konservasi Indonesia, Sorong, Papua Barat 98417, Indonesia; ^4^ BLUD UPTD Pengelolaan KKP Kepulauan Raja Ampat, Waisai, Papua Barat 98417, Indonesia; ^5^ Elasmobranch and Charismatic Species Program, Konservasi Indonesia, Jakarta 12550, Indonesia; ^6^ School of Veterinary and Life Sciences, Murdoch University, Perth, Western Australia 6150, Australia; ^7^ School of Biological Sciences, University of Auckland, Auckland 1010, New Zealand; ^8^ Department of Statistics, University of Auckland, Auckland 1010, New Zealand; ^9^ Integrated Marine Observing System (IMOS) Animal Tracking Facility, Sydney Institute of Marine Science, Mosman, New South Wales 2088, Australia; ^10^ School of Natural Sciences, Macquarie University, Sydney, New South Wales 2109, Australia

**Keywords:** acoustic telemetry, population connectivity, network analysis, population structure, management

## Abstract

The reef manta ray *Mobula alfredi* is present throughout most island groups that form the Raja Ampat archipelago, Indonesia. The species is protected regionally and nationally and is currently managed as a single homogeneous population within the 6.7 million ha archipelago. However, scientific evidence is currently lacking regarding the spatial connectivity and population structure of *M. alfredi* within this archipelago. Using network analysis and an array of 34 acoustic receivers deployed throughout Raja Ampat between February 2016 and September 2021, we examined the movements of 72 subadult and adult *M. alfredi* tagged in seven regions of Raja Ampat. A total of 1094 *M. alfredi* movements were recorded and were primarily concentrated between nearby receiver stations, highlighting frequent local movements within, and limited long-distance movements between regional acoustic receiver arrays. Network analysis revealed highly connected nodes acting as hubs important for *M. alfredi* movements. A community detection algorithm further indicated clusters within the network. Our results suggest the existence of a metapopulation comprising three demographically and geographically distinct subpopulations within the archipelago. They also reveal the importance of Eagle Rock as a critical node in the *M. alfredi* movement network, justifying the urgent inclusion of this site within the Raja Ampat marine protected area network.

## 1. Introduction

Effective spatial management and conservation of wild fauna require a robust understanding of the structure and movement connectivity of populations [1]. For example, identifying the degree of spatial use overlap between two populations of the same species can provide insights into their reproductive ecology, shared use of key habitats or food resources, or important migratory corridors (e.g. [2–4]), from which tailored management strategies can be drawn. An emerging concept transferred from terrestrial ecosystem research to the marine environment is that of a ‘metapopulation’ [5]. A metapopulation is defined as a set of discrete subpopulations of the same species inhabiting the same general geographical region, between which individuals move through migration and dispersal [6]. Two key assumptions that separate a metapopulation from a single panmictic population are that (i) subpopulations are geographically discrete and (ii) the mixing of individuals between subpopulations is less than that within them [6]. In the marine environment, the metapopulation concept is now commonly used particularly for coral reef fish communities that occupy spatially distinct habitats, as well as for other marine organisms that have limited larval dispersal [7]. For marine megafauna, the metapopulation concept has been considered less relevant owing to the ability of these wide-ranging animals to migrate large distances and the extensive home ranges they are generally assumed to occupy [8]. Nevertheless, many marine populations of conservation concern appear to have a metapopulation structure driven by juvenile dispersal and adult migration (e.g. sharks, sea turtles) [9–11].

The globally threatened reef manta ray *Mobula alfredi* (assessed as VU, vulnerable to extinction, on the IUCN Red List) is widely distributed throughout nearshore pelagic waters of the tropical and subtropical Indo-Pacific (e.g. [12,13]). This highly mobile species exhibits strong site affinity, particularly in isolated parts of its range, such as oceanic island chains [14,15]. The species is also capable of undertaking long-distance movements of several hundreds of kilometres [16–19] (up to 1150 km [20]), and therefore, the metapopulation concept has not generally been considered relevant to this species. However, recent genetic studies have revealed fine-scale genetic differentiation between nearby *M. alfredi* populations. For example, Lassauce *et al*. [21] found strong evidence of genetic structure between *M. alfredi* sampled from three different cleaning station aggregation sites located 110–335 km apart in New Caledonia. Similarly, two genetically distinct *M. alfredi* subpopulations were recently identified in Hawaii between oceanic islands located only 150 km apart, yet separated by waters over 2000 m deep [22]. No matches had previously been identified between photographically identified individuals from these two subpopulations, supporting the idea of distinct subpopulations with no connectivity between the nearby islands [15]. Whitney *et al*. [22] also revealed sex-biased migration patterns, showing strong female philopatry among the two populations. These findings highlight the importance of delineating population structure and distinct ‘management units’ for *M. alfredi* to enable effective management and conservation [23] as well as examining the role of sex-biased dispersal on population connectivity.

**Figure 1.**
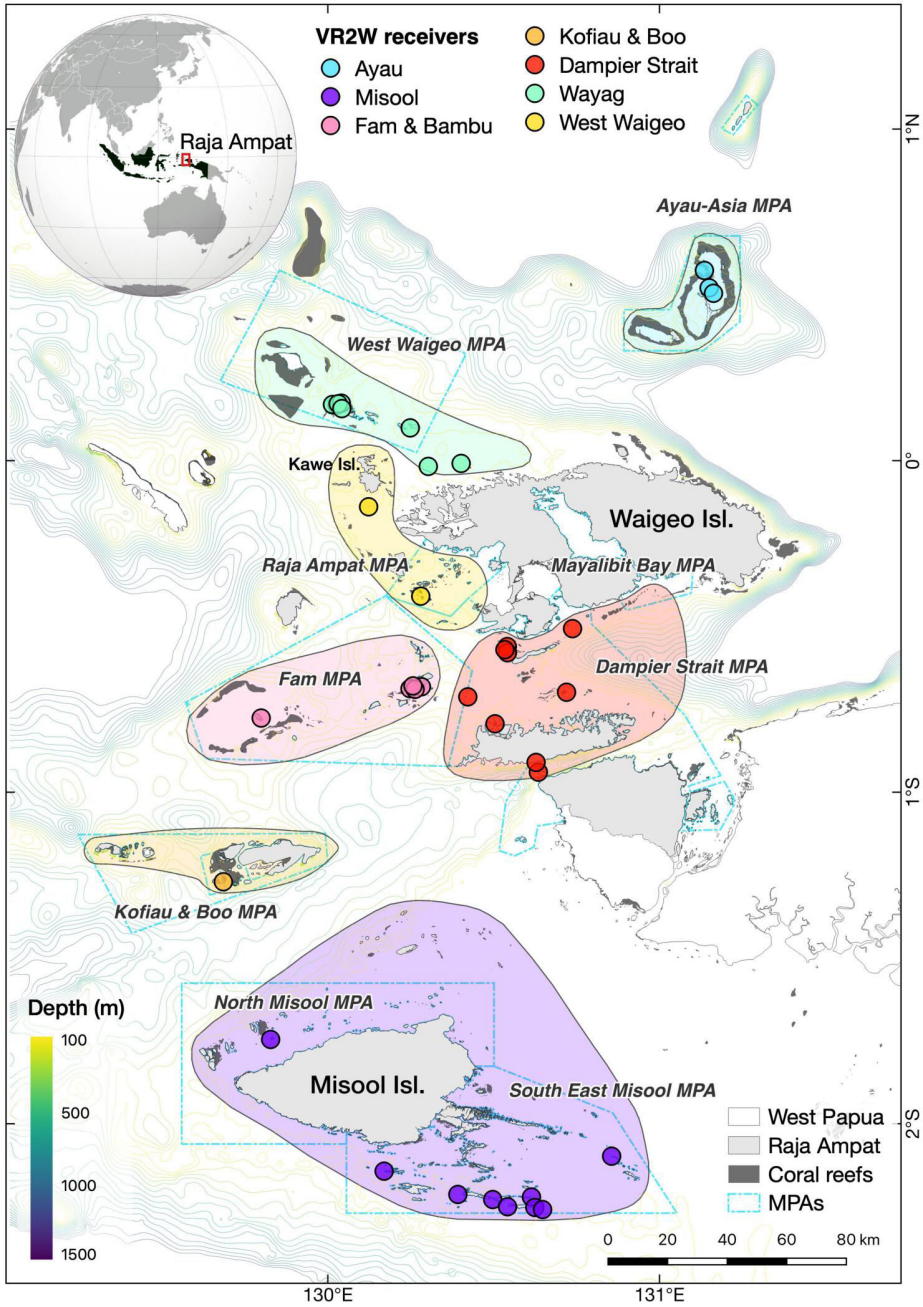
Map of acoustic receivers (coloured circles) deployed throughout the Raja Ampat archipelago. Coloured polygons with solid lines depict the approximate boundaries of island groups (regions) inhabited by hypothesized *M. alfredi* subpopulations. Polygons with blue dash-dotted lines depict MPAs within the Raja Ampat MPA network. Contours show bathymetry throughout the study region.

The Raja Ampat archipelago in eastern Indonesia is home to a large population of *M. alfredi*, with numbers increasing over the past decade [24]. The species has been fully protected in the region since 2012 and is essentially managed by the Raja Ampat Marine Protected Area Management Authority as a single and homogeneous population [25]. Throughout the 6.7 million ha archipelago, the species is distributed unevenly and exhibits high residency and strong affinity to numerous cleaning stations and feeding aggregation sites [19]. Individual *M. alfredi* seasonally migrates between some aggregation sites located along a 130 km corridor through Dampier Strait and West Waigeo [26]. Setyawan *et al*. [19] hypothesized that *M. alfredi* in Raja Ampat might form a metapopulation comprising seven spatially distinct subpopulations inhabiting island groups or regions located 25–125 km apart and separated by waters 800–1400 m deep (figure 1). Each of these island groups or regions, namely Ayau, Wayag Islands, West Waigeo, Dampier Strait, Fam and Bambu, Kofiau and Boo and Misool, was hypothesized to have its own *M. alfredi* subpopulation, between which limited exchange of individuals occurs. A deeper understanding of the connectivity between these hypothesized subpopulations is necessary to support this theory and improve the effectiveness of conservation management strategies for these vulnerable species in the region.

Metapopulations in the marine environment exhibit limited demographic connectivity between local populations (subpopulations) [8], which can be inferred from the movements of individuals between these subpopulations [27]. Various approaches have been used to assess demographic connectivity in marine environments, including visual observations (i.e. photographic identification), mark-recapture and acoustic telemetry [28,29]. Passive acoustic telemetry, consisting of acoustic transmitters and a stationary network of acoustic receivers deployed at strategic locations, is a powerful tool to inform the presence, residency and habitat use of acoustically tagged animals at these sites and detect movements over a range of spatial scales and for extended periods of time [30–32]. Multi-year use of passive acoustic telemetry has enabled tracking the regional movements of highly migratory species, including *M. alfredi* at their aggregation sites in several regions across the Indo-Pacific [26,33–36].

Passive acoustic telemetry has increasingly been used in combination with network analysis in behavioural and movement ecology studies [37]. The inherent structure of acoustic telemetry data suits the application of network analysis to elucidate the directionality and frequency of movements between sites monitored, with nodes typically denoting acoustic receiver stations and edges representing the movements of tagged animals between receiver stations [38,39]. These combined approaches have been successfully used to reveal the population structure, habitat use and connectivity of marine species, including manta rays and other elasmobranchs, at regional to continental scales and over long periods [29,32,38,40–44].

Here, we examine the spatial connectivity and population structure of manta rays in Raja Ampat using acoustic telemetry. We conducted a network analysis of 5 years (between February 2016 and September 2021) of acoustic telemetry data derived from an array of 34 receivers deployed across the seven regions of interest (i.e. island groups) to identify connectivity patterns as well as key migratory corridors and habitats. We also explored potential sex-biased dispersal and movements of acoustically tagged *M. alfredi* in the region.

## 2. Material and methods

### 2.1. Study area

The Raja Ampat archipelago (0.711°S, 130.407°E) in the Bird’s Head Seascape (BHS), Eastern Indonesia, is home to the country’s largest populations of reef [19] and oceanic *M. birostris* manta rays [45]. Over 70 manta ray aggregation sites are distributed throughout the archipelago, protected by a network of nine marine protected areas (MPAs) that cover a large geographical region of nearly 2 million ha [19] (figure 1). Here, both manta ray species have been fully protected because the Raja Ampat regency government designated the entire archipelago as Southeast Asia’s first shark and ray sanctuary in 2012 [25,46].

The Raja Ampat archipelago is characterized by complex coastlines and bathymetry with shallow shelf (<200 m depth) and deep channel (800–1400 m) habitats. The deep channels naturally isolate several groups of islands such as Misool in the south, the Kofiau and Boo island group in the west and the Ayau atolls in the north of the archipelago (figure 1). Primary productivity in the Raja Ampat archipelago is affected by upwellings, occurring during the southeast monsoon in several regions, including the Dampier Strait, Bougainville Strait (in the northwest of Waigeo Island) and southeast Misool [47,48].

### 2.2. Data collection

#### 2.2.1. Transmitter deployments

We deployed V16-5H acoustic transmitters (Innovasea, Halifax, CA), operating at 69 kHz frequency and transmitting pings randomly every 60–130 s, on 117 individual *M. alfredi* throughout the study region. The preparation (i.e. coating and tether length) and deployment of all acoustic transmitter tags followed established procedures used in similar studies in Raja Ampat [26,49]. Each transmitter was attached to a titanium dart with a 12 cm long stainless steel tether coated with heat-shrink tubing. All acoustic transmitters were coated with a non-toxic silicone-based Propspeed™ ablative coating to prevent fouling of the transmitters.

Prior to transmitter deployment, each individual *M. alfredi* was photo-identified and sexed whenever possible. The sex of each individual *M. alfredi* was determined by the presence (male) and absence (female) of claspers on the pelvic fins [50,51]. The disc width (DW) of each individual *M. alfredi* was also visually estimated. The identification photographs and sighting information of photo-identified individuals were then entered into a comprehensive BHS *M. alfredi* sighting database [19]. All acoustically tagged *M. alfredi* were subadults and adults with DW larger than 2.4 m, based on the classification described by Setyawan *et al*. [19].

Each transmitter was deployed externally on *M. alfredi* while free diving or SCUBA diving. We used a pole spear to insert the dart tip into the dorsum of each individual *M. alfredi* in the muscle band between either the right or the left pectoral and body cavities. The acoustic transmitters were deployed in five different phases between February 2016 and February 2020 (electronic supplementary material, figure S1) in seven regions across the Raja Ampat archipelago (figure 1). We note that the number of acoustic transmitters deployed in each region was not equal owing to logistical constraints when undertaking fieldwork in this remote region. Of all transmitters deployed, 36 were deployed in Dampier Strait, 28 in West Waigeo, 28 in Misool, 13 in Fam and Bambu, 6 in Ayau, 4 in Kofiau and Boo and 2 in Wayag (electronic supplementary material, figure S1 and table S1).

#### 2.2.2. Acoustic receiver deployments

To document the presence of acoustically tagged animals at sites of interest, we deployed an array of 34 VR2W-69 kHz acoustic receivers (Innovasea, Canada) across the Raja Ampat archipelago (figure 1) between February 2016 and September 2021. These acoustic receivers were deployed in regions inhabited by the seven hypothesized *M. alfredi* subpopulations [19]: Ayau (*n* = 3), Wayag (*n* = 4), West Waigeo (*n* = 2), Dampier Strait (*n* = 10), Fam and Bambu (*n* = 5), Kofiau and Boo (*n* = 1) and Misool (*n* = 9). Each acoustic receiver, approximately 2 m above the substrate, was securely cable-tied to buoyed moorings that were attached to the substrate [26]. To optimize acoustic detections, we strategically deployed the acoustic receivers within 150 m of *M. alfredi* feeding and cleaning sites or other known aggregation sites based on the results of range tests conducted in two previous studies in Raja Ampat using the same acoustic receiver and transmitter specification which suggested 150–200 m as a maximum distance for reliable acoustic detections in this environment [24,26]. These acoustic receivers were maintained and downloaded every 6 months.

The acoustic receiver array in each region varied from 1 to 10 receivers (figure 1). The deployment periods of these acoustic receivers also varied (electronic supplementary material, figure S2) owing to several factors, including access to remote sites or theft or damage to receivers. Unfortunately, the logistical difficulties of monitoring and replacing stolen or damaged receivers in the remote archipelago led to some notable data gaps at some sites. Given the difference in the number of aggregation sites identified in each region as well as logistical and financial constraints, the acoustic receiver array in each of these regions was not equally dense (figure 1). Variations in the density of the acoustic receiver array and the number of transmitters deployed in each region, as stated in §2.2.1, could potentially affect the level of connectivity between receiver stations within each region and between regions.

### 2.3. Data analyses

#### 2.3.1. Passive acoustic telemetry data

Detection data collected by acoustic receivers for all tagged *M. alfredi* were extracted via the VUE software and recorded as a timestamped log of transmitter IDs detected by acoustic receivers deployed at 34 stations across the study region (figure 1, electronic supplementary material, figure S3). False-positive detections were removed by filtering detection data for active transmitters. Detections recorded prior to tagging owing to the handling of transmitters by the tagger were also removed. The resulting data consisted of transmitter IDs, timestamps of detections, receiver metadata (e.g. geographic coordinates, station category) and transmitter metadata (tagging time and location). To calculate the number of movements of tagged *M. alfredi* between receiver stations, the filtered data were then analysed to extract residence and non-residence events using the ‘RunResidenceExtraction’ function in the ‘VTrack’ package v. 2.10 [52]. A residency event was recorded when one detection from a tagged *M. alfredi* was detected, and it was terminated when either the tagged *M. alfredi* was detected at another receiver station or was not detected at any receiver station within 60 min. For the subsequent analysis, however, only non-residence events were reported as we focused on the movements of acoustically tagged *M. alfredi* between receiver stations instead of their residency at receiver stations. We then filtered the non-residence data for non-residence events involving two different receiver stations. All data filtration and analysis were performed in the R environment [53].

#### 2.3.2. Network analysis

Movement networks were constructed to assess the spatial connectivity of *M. alfredi* between the receiver stations (i.e. monitored sites) throughout Raja Ampat. The movement networks were generated to explore the movement and dispersal patterns of *M. alfredi* tagged in each region. Each movement network consisted of nodes representing receiver stations and edges denoting *M. alfredi* movements (i.e. an animal carrying a specific transmitter ID being detected consecutively at two distinct sites) recorded between these nodes. Edges were weighted based on the proportion of movements recorded during the tracking period. All movement networks were constructed and plotted in both geographic coordinate and multidimensional scale layouts using the ‘igraph’ package [54].

To understand the structure of movement networks, each network was measured for its network-level metrics and node-level metrics. Network-level metrics were measured to understand the patterns of connectivity between all nodes and edges in the network [55]. These metrics consisted of eight measures, including (i) the number of all nodes within the network, (ii) the number of nodes within regions where tagging occurred, (iii) the number of connected nodes, (iv) the number of edges between each pair of nodes, (v) the total number of movements between two nodes across all animals, (vi) edge density (proportion of existing edges out of a total number of possible edges in the network [32]), (vii) average path length (APL; mean length of the shortest path connecting all nodes in the network [55]), and (viii) diameter (longest path between any pair of nodes within the network, indicating the network size [56]) (table 1).

**Table 1.** Network-level metrics of centrality for the observed *M. alfredi* movements in Raja Ampat between February 2016 and September 2021. N nodes (in network) = the total number of nodes in the network; N nodes (in each tagging region) = the number of nodes in the tagging region; N nodes connected = the total number of nodes in the Raja Ampat network that are connected by edge(s); N edges = the total number of edges connecting two nodes in the network; N movement = the total number of movements made by individuals tagged in each respective tagging region; tagging regions = the regions where the acoustic transmitters were deployed.

network metrics	Raja Ampat (receiver station level)	movement networks based on tagging regions						
		Ayau	Dampier Strait	Fam and Bambu	Kofiau and Boo	Misool	Wayag	West Waigeo
N nodes (in Raja Ampat)	34	34	34	34	34	34	34	34
N nodes (in tagging region)	N/A	3	10	5	1	9	4	2
N nodes connected (in Raja Ampat)	32	3	22	5	2	10	2	9
N edges	131	6	74	6	2	43	1	20
N movements	1094	46	288	7	2	625	1	125
edge density	0.117	0.005	0.066	0.005	0.002	0.038	0.001	0.018
average path length	2.71	1	2.73	1.83	1	1.51	1	1.98
diameter	6	1	6	4	1	3	1	4

At the node level, centrality measures (node-level metrics), which were determined from the level of connectivity between nodes either directly or indirectly via other nodes, were calculated for each network to describe the relative importance of a node (i.e. manta ray aggregation site) and the influence of nodes on the overall structure of each movement network [57]. We calculated six centrality measures, including (i) in-degree centrality, (ii) out-degree centrality, (iii) degree centrality, (iv) betweenness, (v) closeness, and (vi) eigenvector (table 1). In-degree and out-degree represent the number of neighbours for each node with incoming and outgoing edges, respectively [58]. Degree centrality shows the number of all edges connected to a node, which is the sum of in-degree and out-degree [59]. Betweenness demonstrates the number of shortest paths crossing through a node, which indicates how much a receiver station was involved in the movements of *M. alfredi* [60]. Closeness calculates the average distance from a node to other nodes, showing how central the position of a node is within the network. Eigenvector indicates how important a node is within a network by considering the degree of centrality of other nodes connected to this node [59].

To determine if the space use of *M. alfredi* within the acoustic receiver array occurred in a non-random manner, the network was tested for non-random movements of acoustically tagged animals using edge permutation [57]. A network with a structure typically has a longer APL than a random network with the same number of nodes and edges [61]. The null hypothesis was that each node in the observed network had the same probability of being connected to other nodes through the movements of *M. alfredi* despite its distance to another; therefore, the observed network would have a similar APL to a random network. The edge permutation was performed based on observed movements between nodes in the network, with 10 000 bootstrap iterations. Edges from the observed network were shuffled randomly, and then new networks were generated using the same degree distribution as the observed network using the *degree.sequence.game* function from the igraph package [54]. Following this, the distribution of APL values obtained from these newly permuted networks was then compared with the APL of the observed network. A *P*-value was calculated based on a one-tailed test to examine if the probability of the observed APL fell within the distribution of APL values from the permuted networks.

Following this non-random test, we used a community detection algorithm based on modularity [62] to identify community structure within the acoustic receiver array network and determine clusters consisting of densely connected nodes (acoustic receiver stations) with lesser connectivity across clusters [63]. A positive value of modularity indicates the possible presence of community structure within the network, and a modularity of 0.3 or larger suggests a good division within the network to generate clusters [62,64]. The analysis was undertaken using the *cluster_optimal* function in the igraph package by including edge weights representing the number of *M. alfredi* movements within pairs of nodes (acoustic receiver stations) [54]. Two nodes (receiver stations in Uranie and North Misool) were removed from this analysis, as they were unconnected to other nodes, leaving 32 of 34 nodes.

Finally, to assess differences in movement patterns between female and male *M. alfredi*, the total movements made between pairs of receiver stations were examined for males and females separately. A Shapiro–Wilk normality test was used to test if the data were normally distributed, and an *F*-test was used to test for homogeneity in the variances of the data, before applying an unpaired two-sample *t*‐test to determine if there were any differences between females and males in each of the two measures. All statistical significances for hypothesis tests were reported based on Muff *et al.* [65].

## 3. Results

### 3.1. Passive acoustic tracking

Passive acoustic tracking of *M. alfredi* in the Raja Ampat archipelago was conducted between February 2016 and September 2021. During this period, a total of 60 500 acoustic detections were recorded by 32 of the 34 receivers deployed across the Raja Ampat archipelago (figure 1). The total number of days each individual *M. alfredi* was detected ranged from 1 to 194 days (mean ± s.d. = 30 ± 41 days). Of the 117 transmitters deployed, 94 tagged individuals (80%) were detected at least once by acoustic receivers in the array (electronic supplementary material, figure S3). Of these 94 individuals, 72 were detected by two or more receiver stations. Movements between receiver stations were then examined for these 72 *M. alfredi* (44 females, 27 males and 1 unsexed individual).

A total of 1094 movements were recorded (table 1), consisting of 777 movements by females, 315 movements by males and 2 movements by the unsexed individual. Among the 72 individuals detected by two or more receivers, the average of total movements was 15 (s.d. = 26). To examine the potential sex-biased movements and dispersal of acoustically tagged *M. alfredi*, we compared the average of total movements and mean direct distance travelled between sexes, respectively. Given the variability of receiver deployments and tagging effort that was unevenly distributed in both sexes at each site, we therefore examined only the movements of animals tagged at Manta Ridge in Dampier Strait, where the number of females (*n* = 10) and males (*n* = 7) detected by at least two receivers was similar and this region had high network-level metrics (i.e. total number of edges and movements, edge density, APL and diameter; table 1). Between sexes, females (mean ± s.d. = 10 ± 4 movements) moved more frequently than males (mean ± s.d. = 7 ± 5 movements). We determined that an unpaired two-sample *t*‐test is appropriate to test for differences between males and females in terms of their average total number of movements, because a Shapiro–Wilk test did not provide evidence to suggest the data were not normally distributed (*p* > 0.05), and an *F*-test did not indicate differences in variance between sexes (*p* > 0.05). The *t*‐test revealed that there was no evidence (*p* = 0.355) of a difference in the average of total movements made by females (median = 10 movements) and males (median = 6 movements) (electronic supplementary material, figure S4). In terms of the average direct distance travelled by tagged *M. alfredi* at Manta Ridge, the average was similar between females (mean ± s.d. = 28.5 ± 16.3 km) and males (mean ± s.d. = 28.2 ± 18.1 km). We determined that an unpaired two-sample *t*‐test is appropriate to test for differences between males and females in terms of their average direct distance travelled by the tagged *M. alfredi* because a Shapiro–Wilk test did not provide evidence to suggest the data were not normally distributed (*p* > 0.05), and an *F*-test did not indicate differences in variance between sexes (*p* > 0.05). The *t*‐test revealed that there was no evidence (*p* = 0.980) of a difference in the average total direct distance travelled by females (median = 28 km) and males (median = 24 km; electronic supplementary material, figure S4).

### 3.2. Movements between acoustic receiver stations

**Figure 2.**
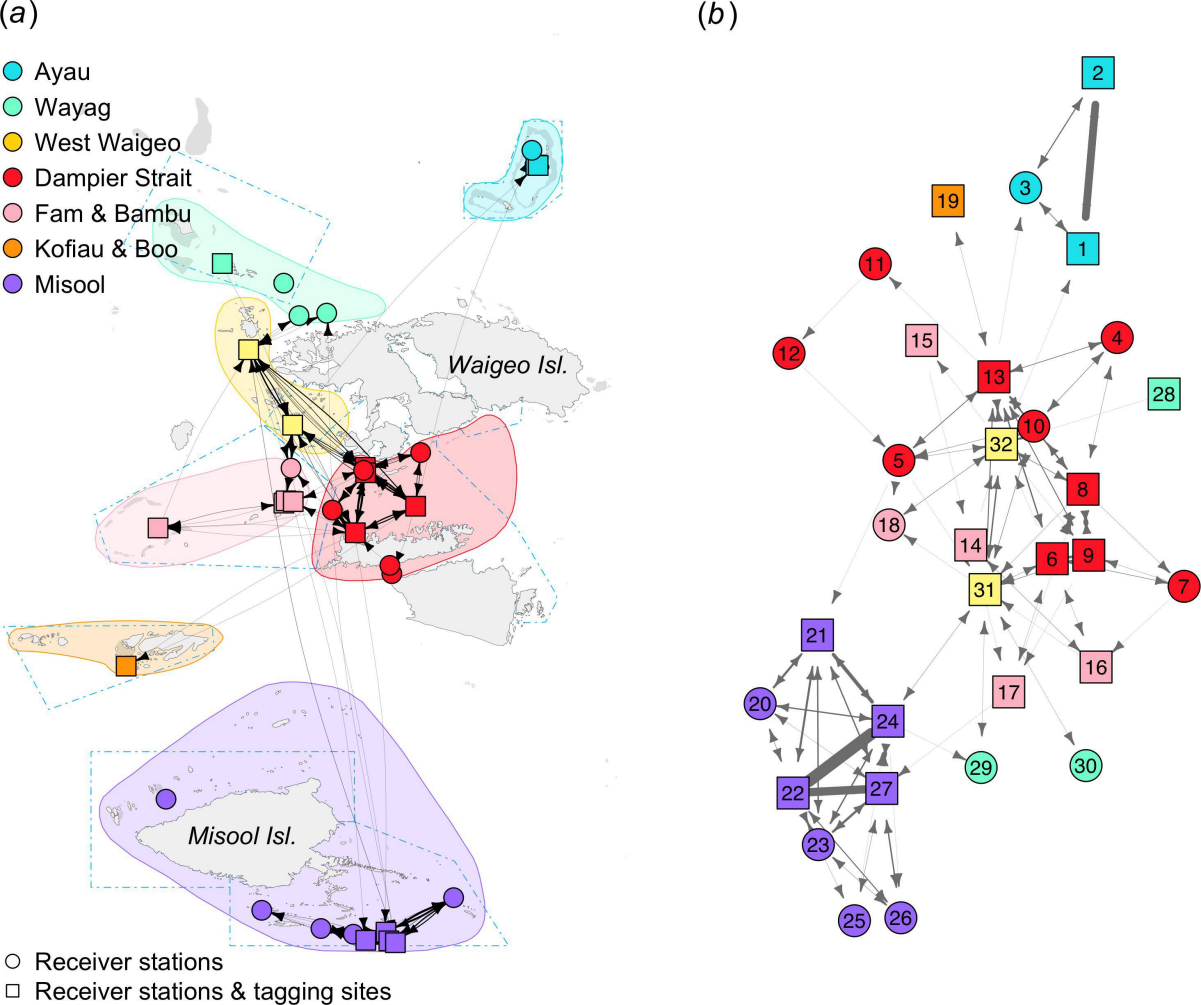
Movement networks for *M. alfredi* acoustically tracked across Raja Ampat between February 2016 and September 2021 displayed using geographic coordinates (*a*) and a multidimensional scaling layout (*b*). Nodes (coloured circles and squares) symbolize either receiver stations or both tagging sites and receiver stations. Edges (grey lines) represent the movements of *M. alfredi* between the nodes. Arrows indicate the direction of movements. The thickness of the edges represents the frequency of movements between nodes (the thicker the lines, the more frequent movements occur between two connected nodes). Blue polygons with blue dash-dotted lines depict MPAs within the Raja Ampat MPA network. Node labels in (*b*): 1. Ayau Besar Cleaning Station, 2. Ayau Besar Feeding Ground, 3. Ayau Besar Lagoon Entrance, 4. Blue Magic, 5. Dayan, 6. Dayan Cleaning Station, 7. Karang Bata, 8. Manta Ridge, 9. Manta Sandy, 10. Pasir Timbul, 11. Sagawin, 12. South Batanta, 13. Wai, 14. Andau Besar, 15. Andau Kecil, 16. Bambu, 17. Meoskor, 18. Penemu, 19. Kofiau, 20. Daram Andiamo, 21. Devil’s Kitchen, 22. Eagle’s Nest, 23. Fish Mount, 24. Magic Mountain, 25. Pelee’s Playground, 26. Rats Reef, 27. Southwest Batbitim, 28. Main Lagoon Entrance, 29. Sepatu, 30. Seprang, 31. Eagle Rock, 32. Yefnabi Kecil.

The movement network of 72 tagged *M. alfredi* was constructed from 34 nodes (receiver stations) and 131 edges (figure 2), consisting of a total of 1094 movements between these nodes (table 1). All nodes were connected, except for the two receiver stations of North Misool in Misool and Uranie in Wayag. At the regional level, nine stations within the Misool regional receiver array, eight of which were closely located in Southeast Misool MPA (figure 1), seemed to be closely connected with each other, and frequent *M. alfredi* movements were recorded primarily between receivers at Magic Mountain, Eagle’s Nest and Southwest Batbitim. Similar to Misool, three receiver stations in Ayau were closely connected and grouped together, with frequent movements of *M. alfredi* between two receivers at a cleaning station and a feeding ground. In contrast, the 17 receiver stations in central Raja Ampat (Dampier Strait, Fam and West Waigeo), where more than half of the transmitters were deployed, were all connected with the others with various degrees of movements between them. The single receiver station in Kofiau, where only four transmitters were deployed in the region, was quite isolated from other receiver stations in the network and was only connected with the Wai receiver station (Dampier Strait). Furthermore, three receiver stations in the Wayag region, where only two transmitters were deployed, were connected with both receiver stations in the West Waigeo region and with the acoustic receiver at Magic Mountain in Misool.

The majority (92.5%) of the 1094 recorded movements occurred between receiver stations within each regional receiver array, especially in Misool, Ayau and Dampier Strait, where receivers in these regions were geographically located close to each other (electronic supplementary material, table S2). In Misool, four receiver stations (Magic Mountain, Eagle’s Nest, Southwest Batbitim and Devil’s Kitchen) contributing to 41% of the total movements recorded were located within a maximum of 11.8 km from one another. In Ayau, two receiver stations in Ayau (Ayau Besar Cleaning Station and Ayau Besar Feeding Ground), which are located 930 m apart, contributed to 11% of the total movements recorded. In Dampier Strait, two receiver stations (Manta Ridge and Manta Sandy) were located 2.2 km apart and contributed to 7% of the total movements recorded.

Electronic supplementary material, table S3 lists the centrality measures of the 34 receiver stations in the Raja Ampat network (figure 2); the eight receivers showing the highest degree centrality measures are located within the Dampier Strait (i.e. Wai, Dayan Cleaning Station, Manta Ridge), West Waigeo (i.e. Eagle Rock, Yefnabi Kecil) and Misool (i.e. Magic Mountain, Southwest Batbitim, Eagle’s Nest) regional receiver arrays. Moreover, most of these receiver stations had higher values of betweenness, closeness and eigenvector, which emphasized the relative importance of these receiver stations compared to others in the Raja Ampat receiver array network. Eagle Rock in West Waigeo and Wai in Dampier Strait recorded the highest degree centrality values, and Eagle Rock had a substantially higher betweenness value than all other receiver stations, indicating that Eagle Rock was connected to many other receiver stations and highly central and influential in the regional movements of *M. alfredi* in Raja Ampat.

### 3.3. Detecting structure in the movement network

**Figure 3.**
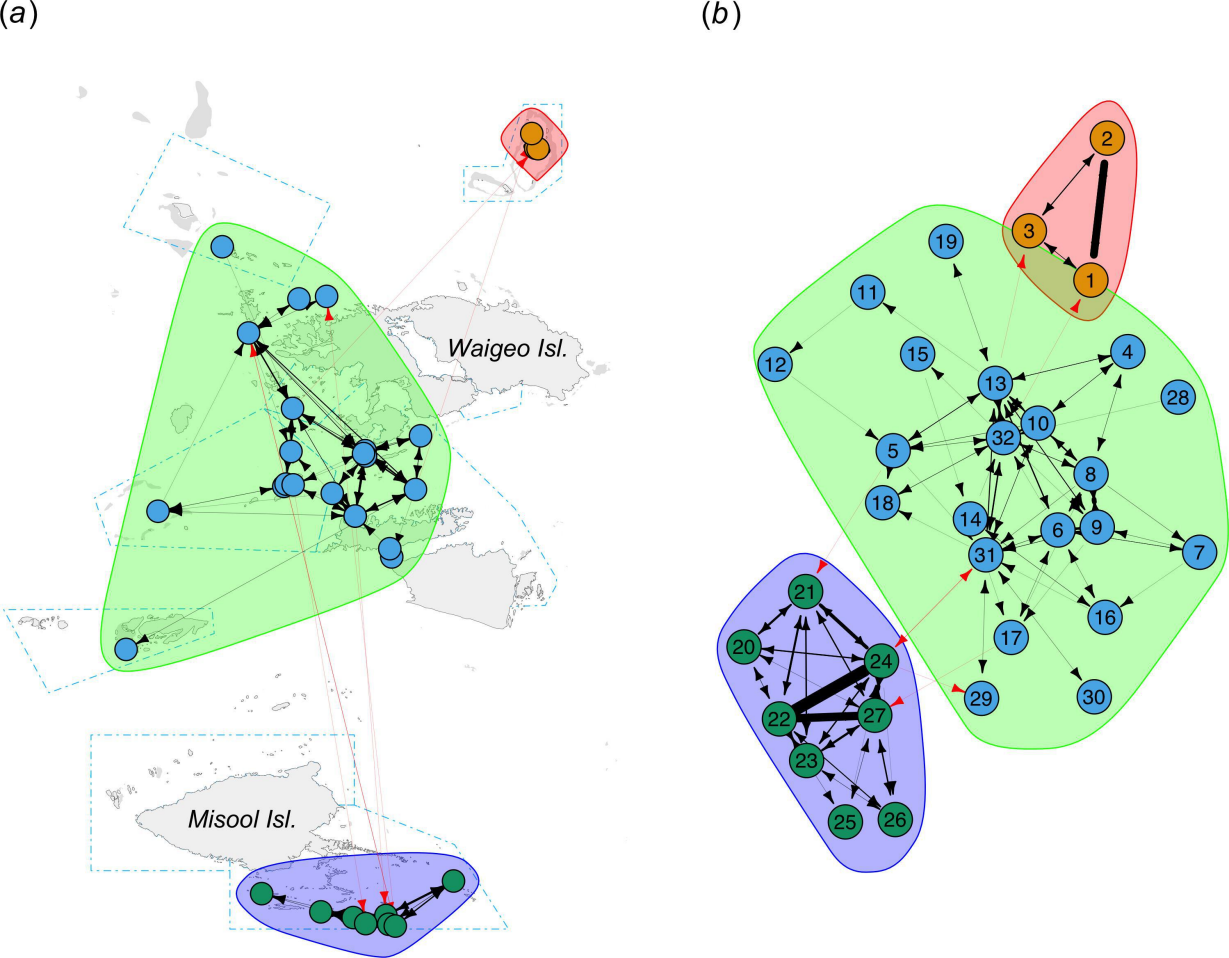
Movement network for Raja Ampat *M. alfredi* showing cluster-based community structure displayed using geographic coordinates (*a*) and a multidimensional scaling layout (*b*). Nodes (coloured circles) symbolize acoustic receiver stations. Edges represent the movements of *M. alfredi* between the nodes. Black arrows indicate the direction of movements within the clusters, while red arrows represent movements between clusters or subpopulations. The thickness of the edges represents the frequency of movements between nodes (the thicker the lines, the more frequent movements occur between two connected nodes). The colours of nodes and clusters represent different *M. alfredi* subpopulations identified by the analysis. Blue polygons with blue dash-dotted lines depict MPAs within the Raja Ampat MPA network. Node labels in (*b*): 1. Ayau Besar Cleaning Station, 2. Ayau Besar Feeding Ground, 3. Ayau Besar Lagoon Entrance, 4. Blue Magic, 5. Dayan, 6. Dayan Cleaning Station, 7. Karang Bata, 8. Manta Ridge, 9. Manta Sandy, 10. Pasir Timbul, 11. Sagawin, 12. South Batanta, 13. Wai, 14. Andau Besar, 15. Andau Kecil, 16. Bambu, 17. Meoskor, 18. Penemu, 19. Kofiau, 20. Daram Andiamo, 21. Devil’s Kitchen, 22. Eagle’s Nest, 23. Fish Mount, 24. Magic Mountain, 25. Pelee’s Playground, 26. Rats Reef, 27. Southwest Batbitim, 28. Main Lagoon Entrance, 29. Sepatu, 30. Seprang, 31. Eagle Rock, 32. Yefnabi Kecil.

A non-random test suggested there was strong evidence (*p* < 0.001) that the APL of the observed network (2.708) was higher than that we would expect from random networks (electronic supplementary material, figure S5), suggesting that the movements of acoustically tracked *M. alfredi* were non-random and thus there was a structure within the movement network. A community detection algorithm on 32 of 34 nodes yielded a positive modularity score of 0.558, indicating the presence of structure in the network in the form of three distinct node clusters. The algorithm revealed that the 32 nodes were grouped into three different clusters representing different regions, consisting of Ayau, Misool and central Raja Ampat (figure 3). The first cluster consisted of all three receiver stations located in Ayau, and similarly, all receiver stations in Misool were classified into another tight cluster. Interestingly, all receiver stations deployed in Wayag, West Waigeo, Fam and Bambu, Dampier Strait and Kofiau and Boo were classified into a large single cluster.

### 3.4. Movements of *M. alfredi* acoustically tagged in each region

**Figure 4.**
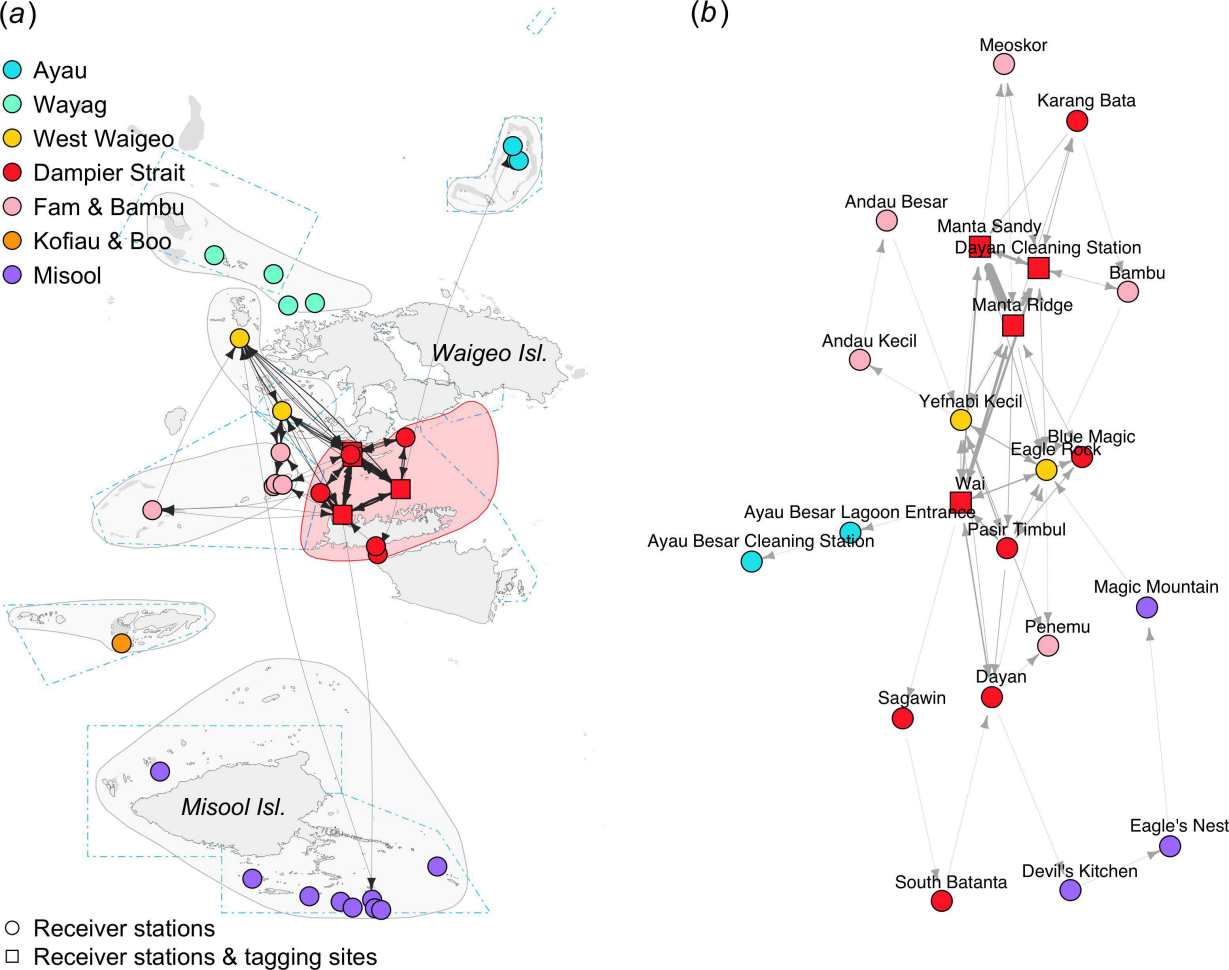
Movement networks for *M. alfredi* acoustically tagged in the Dampier Strait region between February 2016 and February 2020. Geographic coordinate layout (*a*). Multidimensional scale layout (*b*).

Of the 36 individuals tagged in the Dampier Strait, 29 were detected by at least two receiver stations (electronic supplementary material, table S1), resulting in 288 movements between receiver stations (table 1). The movement network for *M. alfredi* tagged in the Dampier Strait region showed high connectivity between four receiver stations where tagging occurred: Manta Ridge, Manta Sandy, Wai and Dayan Cleaning Station (figure 4). These receiver stations also acted as hubs connecting Dampier Strait with other regional receiver arrays in Fam and Bambu, West Waigeo, Misool and Ayau (electronic supplementary material, table S2). The edge density for the movement network of individuals tagged in Dampier Strait was the highest of all movement networks based on tagging region, suggesting substantially more frequent local movements within the Dampier Strait regional receiver array (table 1).

**Figure 5.**
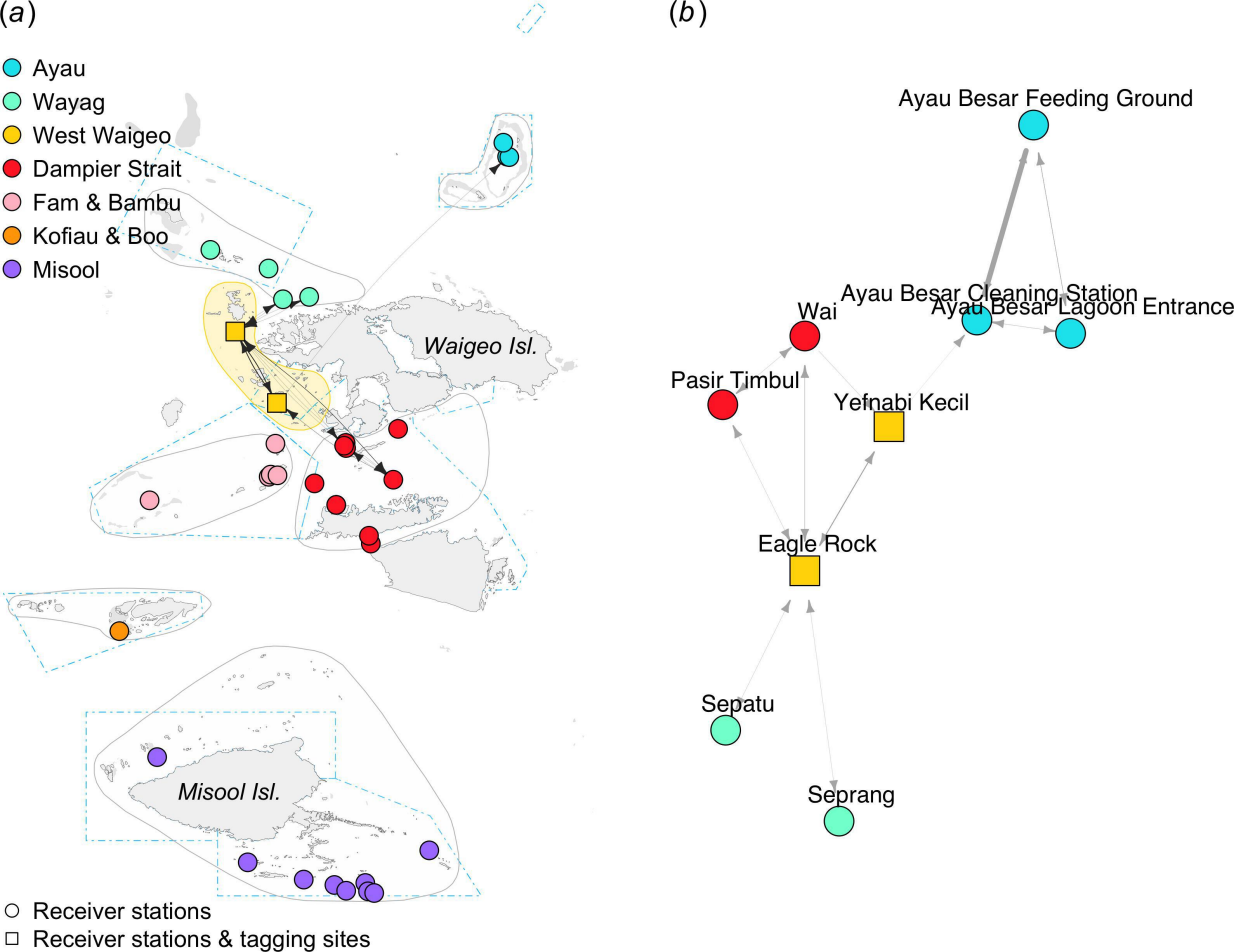
Movement networks for *M. alfredi* were acoustically tagged in the West Waigeo region between February 2016 and February 2020. Geographic coordinate layout (*a*). Multidimensional scale layout (*b*).

The 28 *M. alfredi* acoustically tagged in the West Waigeo region were recorded 125 movements, including those to two neighbouring regions (Dampier Strait and Wayag) and to the distant Ayau region (table 1, figure 5). Most movements recorded in the Ayau region were from an individual tagged at Yefnabi Kecil in West Waigeo. Several movements were also recorded between the only two receiver stations in West Waigeo (Eagle Rock and Yefnabi Kecil).

**Figure 6.**
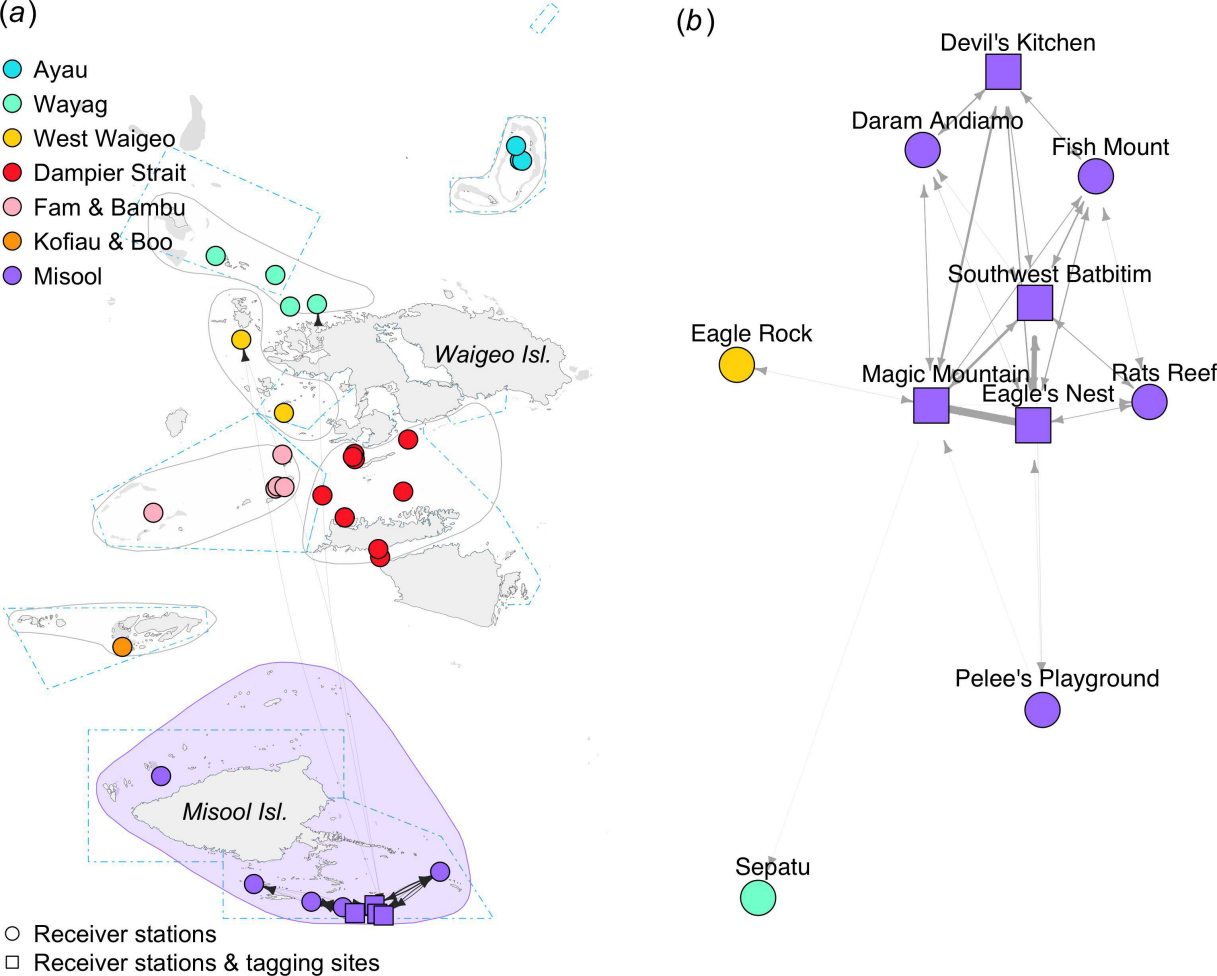
Movement networks for *M. alfredi* acoustically tagged in the Misool region between February 2016 and February 2020. Geographic coordinate layout (*a*). Multidimensional scale layout (*b*).

The movement network of *M. alfredi* tagged in Misool was constructed from 10 connected nodes, mainly from the Misool regional receiver array (figure 6). Of the 28 tagged *M. alfredi*, 24 were detected by two or more receiver stations, resulting in 625 movements (57% of total movements in the study) that were recorded mainly within the Misool regional receiver array (table 1). Two movements were detected between Magic Mountain in Misool and Eagle Rock in West Waigeo, which are located ~240 km apart. Another relatively long-distance movement was recorded from Magic Mountain to Sepatu in the Wayag region, located ~275 km away.

**Figure 7.**
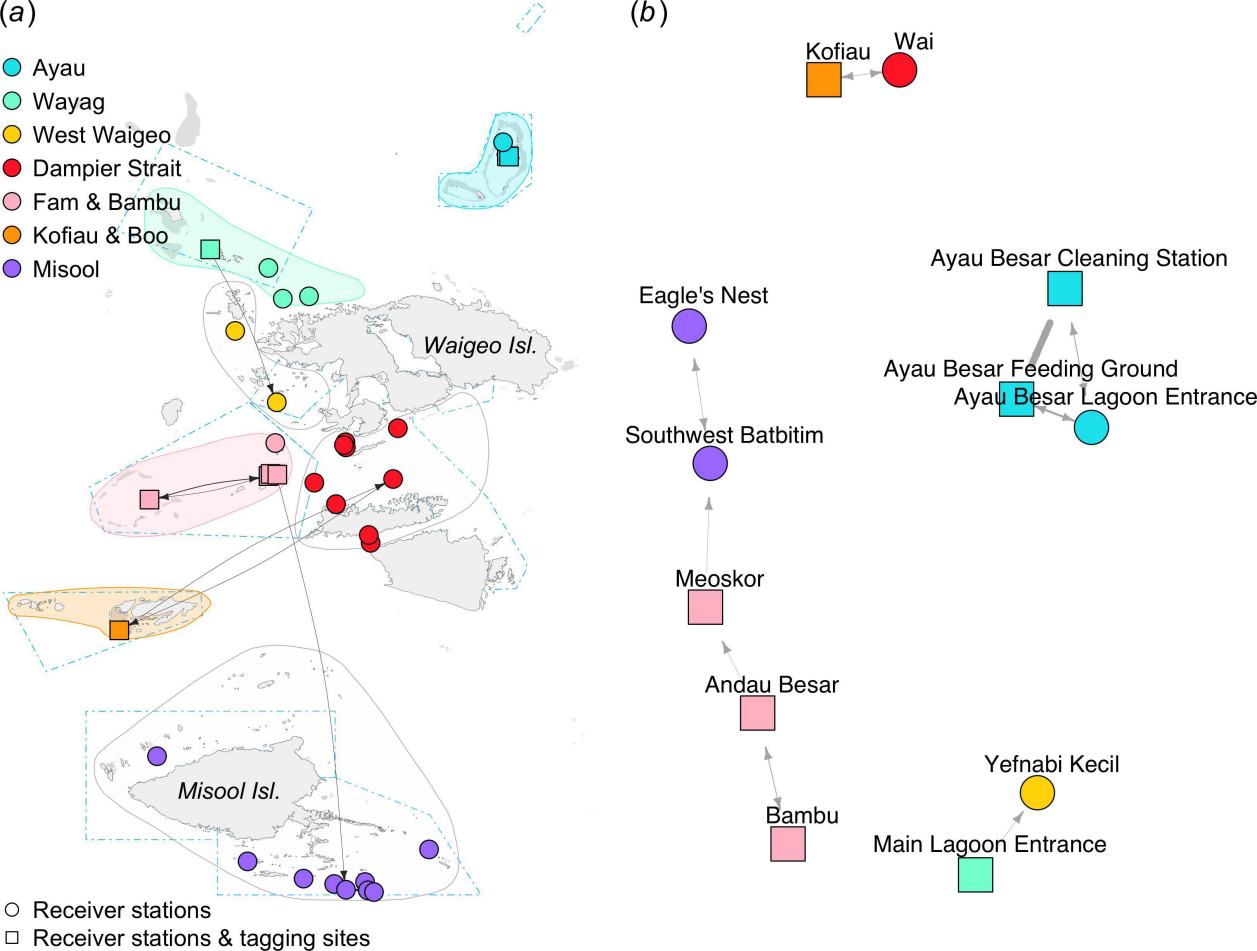
Movement networks for *M. alfredi* acoustically tagged in four regions (Ayau, Wayag, Fam and Bambu and Kofiau and Boo) between February 2016 and February 2020. Geographic coordinate layout (*a*) and multidimensional scale layout (*b*).

Of the 13 individuals tagged using acoustic transmitters in the Fam and Bambu region, only three were detected by two or more receiver stations. Several movements were detected by receiver stations within the Fam and Bambu regional array, including those between Bambu and Andau Besar (figure 7). One receiver station (Meoskor) acted as a hub connecting Fam and Bambu manta rays with those in the Misool region via the Southwest Batbitim receiver station, ~175 km away to the south. In the Wayag region, an individual tagged at the Main Lagoon Entrance moved to Yefnabi Kecil in West Waigeo. Movements were also detected between receiver stations in Kofiau and Wai by an individual tagged in Kofiau. Interestingly, the movements of individuals tagged in Ayau were only recorded by the three Ayau receiver stations.

## 4. Discussion

### 4.1. *M. alfredi* metapopulation and movements between subpopulations

Our combined approach using passive acoustic telemetry and spatial network analysis enabled further investigation of the degree of connectivity between key *M. alfredi* aggregation sites throughout the study area [37,39]. This study provides strong evidence that *M. alfredi* in the Raja Ampat archipelago forms a metapopulation consisting of three distinct subpopulations inhabiting the Ayau, Misool and central Raja Ampat regions. Network analysis of an extensive acoustic telemetry dataset revealed that these subpopulations are geographically discrete, with limited movements detected between these regions. These results fulfil the two key requirements of Akçakaya *et al*.’s [6] metapopulation definition: (i) the subpopulations are geographically discrete and (ii) the mixing of individuals between the subpopulations is less than that within them. Previously, Setyawan *et al*. [19] subdivided the central Raja Ampat region into five separate hypothesized subpopulations: Kofiau and Boo and four subpopulations in northwestern Raja Ampat (Dampier Strait, West Waigeo, Fam and Bambu and Wayag). Based on the findings in this study, it appears that these five hypothesized subpopulations show enough mixing to warrant their merging into a single large subpopulation.

Variability in the acoustic transmitter and receiver deployments in each region could affect the number of movements and hence the level of connectivity between the hypothesized *M. alfredi* subpopulations. Movements were likely detected more (or less) frequently in regions where more (or less) transmitters and receivers were deployed, which potentially impacted the detection of structure in the movement network (see §4.3). Although the community detection algorithm suggested that the manta rays in the Kofiau and Boo island group should be considered as part of this single large subpopulation in northwestern Raja Ampat, our analysis of the connectivity of this particular subregion was significantly hindered by both the small number of transmitters deployed in the region and especially by having only a single acoustic receiver present for detections. One of the four individuals tagged in Kofiau moved outside of the region (to Wai in Dampier Strait, then subsequently back to Kofiau), which was sufficient for the network analysis to group Kofiau within the northwestern Raja Ampat subpopulation. Nonetheless, we note that none of the other manta rays tagged within the study moved from or to Kofiau, and other lines of evidence suggest that Kofiau manta rays are largely isolated. A single individual reef manta ray satellite tagged in Kofiau likewise showed a limited home range restricted to the Kofiau region [66]. As of 8 August 2023, our BHS *M. alfredi* sighting database [19], containing verified photographic records of 1834 individuals from 8542 sightings from April 2003, revealed movements of only two individuals between Kofiau and Dampier Straits. These two individuals were frequently sighted in Dampier Strait and were then resighted only once in Kofiau before being resighted on a few occasions in Dampier Strait. The Kofiau and Boo island group is moreover separated by deep water (500–900 m) from all adjacent regions in Raja Ampat, which lends further support to the hypothesis that it might best be considered an isolated subpopulation of its own. Though we provisionally include Kofiau and Boo in the northwestern Raja Ampat subpopulation based on the results of this study, further investigation of the connectivity of the manta rays in this region is clearly warranted in the future to untangle these conflicting lines of evidence.

The network analysis demonstrated frequent movements and high connectivity between acoustic receiver stations within the suggested subpopulations, and limited movements and low connectivity between them. Individuals in Ayau and Misool exhibited frequent localized movements between receiver stations within their respective regional arrays and displayed little connectivity with the other subpopulations in northwestern Raja Ampat. In contrast, substantial connectivity, and frequent local and regional movements between these cleaning and foraging aggregation sites in Dampier Strait, Fam Islands and West Waigeo, were previously observed using individual photographic identification techniques and passive acoustic telemetry [19,26]. This suggests that *M. alfredi* in northwestern Raja Ampat is likely panmictic and should be considered as a single large subpopulation. Some of the aggregation sites located between Dampier Strait and Wayag Islands may form an important seasonal *M. alfredi* migration corridor as revealed by passive acoustic telemetry [26]. Similarly, satellite tracking of large individuals revealed some degree of overlap in the home ranges of *M. alfredi* tagged in the Dampier Strait and West Waigeo regions but no overlap with the home range of those tagged in other regions (Kofiau and Boo, Ayau and Misool) [66]. The high degree of movements recorded within the large subpopulation in northwestern Raja Ampat is likely affected by the close proximity (50–120 km) of the island groups between Dampier Strait and Wayag Islands. Similarly, *M. alfredi* at the Komodo Islands, central Indonesia, seasonally moves between aggregation sites along a ~40 km corridor [33]. The relatively shallow bathymetry in Raja Ampat’s northwestern region (figure 1) further facilitates connectivity between aggregation sites. The Dampier Strait–Fam–West Waigeo regions lay on a shallow shelf of only 50–100 m depth, while Wayag and its neighbouring island chain are located on a slightly deeper shelf (~150 m deep).

Despite the species’ ability to migrate to seasonally productive areas located several hundred kilometres away [18,67–69], *M. alfredi* tagged in Ayau, Kofiau and Boo and Misool showed relatively restricted home ranges with only occasional long-distance movements [66]. This is likely explained by several factors, including natural barriers (i.e. deep water) presenting challenges to such frequent movements, as well as sufficient local prey availability and the nearby presence of key cleaning habitats. The remote Ayau, Kofiau and Boo and Misool regions are largely surrounded by deep water, separating them from the shallow shelf around the coast of Waigeo Island (figure 1). Some studies have suggested that deep water (>1000 m depth) acts as the primary barrier to inter-island and long-distance pelagic movements by *M. alfredi* [14,70,71], likely related to the increased risk of exposure to large predators or challenges navigating in open seas. Deep water is believed to be responsible for the limited connectivity between the subpopulations of *M. alfredi* located 150 km apart in Hawaii [15], and between two cleaning station sites in New Caledonia [21]. Ayau is the most isolated region in the Raja Ampat archipelago and is separated by a ~25 km span of 1400 m deep water from the north coast of Waigeo Island. Moreover, no movements were recorded between Kofiau and Boo and Misool despite these regions being only 50 km apart. The 800–900 m deep channel between Kofiau and Boo and Misool likely serves as a barrier to movements of *M. alfredi* between these regions and is an area known for frequent observations of killer whales (*Orcinus orca*), a known predator of manta rays [48]. Several *M. alfredi* movements recorded from Misool to the northwest Raja Ampat region are likely using the relatively shallow shelf (mostly no deeper than 60 m with one 300 m trough in the Sagawin Strait) between Misool and Dampier Strait (~160 km apart) [19]. This enables some individuals to travel relatively long distances while remaining in shallow shelf waters.

Variation in long-distance movements and extended home range suggests that *M. alfredi* may be best described as partial migrants, of which the population consists of resident and migratory individuals [72]. Geographically, this variation is likely influenced by the specific bathymetric profiles of different island and coastal environments. For instance, the island chain of the Lesser Sunda Islands allows some *M. alfredi* to migrate between aggregation sites in Nusa Penida and Komodo [16], situated approximately 400 km apart, while remaining largely in shallow coastal waters. The continuous coastal shelf along eastern Australia allows individuals to move as far as 1150 km without crossing deep water [17,20]. Despite this, individual *M. alfredi* that are partial migrants might undertake occasional long-distance dispersal in search of food, moving over deep water and acting as transient individuals visiting an area for a short period. A female *M. alfredi* recorded in Cocos Island, Costa Rica, was likely to have migrated to this site after crossing extensive deep water [73], noting that the nearest confirmed sighting location was nearly 6000 km away in the Marquesas Islands [74]. This situation does not seem to be the case in Raja Ampat; reliable and sufficient food sources likely eliminate the need for long-distance migration from even the isolated subpopulations, especially Misool and Ayau. Peel *et al.* [75] suggested that island formations comprising atolls or small island groups that are surrounded by or in the vicinity of deep waters often generate zooplankton accumulation through the island-mass effect [76,77] and therefore offer abundant food resources. This factor likely contributes to the strong residency of *M. alfredi* in Ayau and Misool (and potentially Kofiau) and their limited connectivity with the large subpopulation around Waigeo Island in northwestern Raja Ampat.

### 4.2. Key *M. alfredi* aggregation sites and habitats

Node-level metrics derived from the movement network revealed eight receiver stations in the Dampier Strait, West Waigeo and Misool that were well connected with others and had a high degree of centrality, indicating strong site fidelity by wide-ranging animals [57]. Each of these eight receiver stations happens to be located nearby prominent manta ray cleaning stations. Cleaning stations play several crucial roles in the life cycle of manta rays, including serving as the venue for a number of important biological processes (e.g. removing parasites from their skin) and social interactions with other manta rays [50,78]. Visiting cleaning stations that are located in shallow, warm habitats is also likely to physiologically benefit manta rays by increasing metabolic, digestive and gestation rates [79,80]. Over 70 feeding aggregation sites and cleaning stations distributed across Raja Ampat waters [19] support *M. alfredi* philopatric behaviour and seasonal movements influenced by monsoonal prey availability [26,33,69]. These eight aggregation sites distributed in the Dampier Strait, West Waigeo and Misool, appear to play a central role as hubs for the spatial movements and migration of *M. alfredi* in Raja Ampat, are also used as feeding sites (i.e. Eagle Rock and Yefnabi Kecil in West Waigeo, and Wai and Manta Ridge in Dampier Strait) and have been identified as key habitats providing essential services for *M. alfredi* both locally and regionally [19,26,44].

All the nodes playing central roles in the *M. alfredi* movement network are well-protected within the Raja Ampat MPA network [19], except for Eagle Rock, which was identified as a critical node in the *M. alfredi* movement network. We suggest that Eagle Rock should be urgently considered for inclusion in the Raja Ampat MPA network. In the future, it might be worthwhile to assess the impact of habitat loss through removal analysis (e.g. removing a central node like Eagle Rock from a network) on the stability of the movement network [57].

### 4.3. Limitations

Our research has revealed some limitations when using passive acoustic telemetry to investigate metapopulation structure and connectivity between subpopulations. The number of *M. alfredi* tagged in each region and the tracking duration might be insufficient to make inferences at the regional level owing to the variations in individual behaviour identified in several other *M. alfredi* tagging studies [18,36,81]. Sequeira *et al*. [82] showed that a relatively high number of tagged animals is required to acquire meaningful datasets to inform robust studies about marine species’ population structure, habitat use or migratory corridors. Lédée *et al*. [29] also suggested that there is a threshold for the number of tagged individuals required to make inferences at a population level, and the minimum sample size is species-specific depending on various factors (e.g. species behaviour) [32]. The small number of acoustic transmitters deployed in some of our study regions (e.g. Kofiau and Boo) may not have been sufficient to capture the breadth of connectivity patterns in the associated subpopulation. However, while we did not conduct removal analysis to calculate the minimum sample size needed [29], the movements of the 72 *M. alfredi* tracked across the broader region as part of this study conformed well with results obtained for the same population by Setyawan *et al*. [26].

The number of acoustic receivers and array configuration (i.e. number, location and distance) in each region likely influenced our results to some extent. Our study found that the movements of *M. alfredi* between receiver stations were more frequent between those located in closer proximity to each other. This is similar to findings by Perryman *et al*. [44] using smaller acoustic receiver arrays around Manta Ridge, Manta Sandy and Wai in Dampier Strait. Logistical and financial constraints prevented us from having equally dense acoustic receiver arrays in all regions with some regions—the Kofiau and Boo island group, Fam and Ayau—having only one to four receiver stations. Particularly in the Kofiau and Boo island group, the deployment of only a single receiver limited the ability to make inferences about the region as a potential subpopulation, and future passive acoustic telemetry studies in Raja Ampat should ensure at least two or more receivers in each study region.

### 4.4. Future research

Several recent genetic studies have found evidence of significant population structure in *M. alfredi* populations in oceanic island archipelagos, adding further weight to the utility of the metapopulation concept in describing *M. alfredi* population dynamics. In New Caledonia, Lassauce *et al*. [21] found genetic differentiation between *M. alfredi* using three cleaning stations located only 110–335 km apart. While one of these aggregation sites was separated by a 2000 m deep channel from the two other sites, the other two were connected through shallow water and continuous coastal habitats and do not show any obvious barriers to movement. In Hawaii, genetic structuring was found between *M. alfredi* populations from two aggregation sites located only 150 km apart but separated by 2000 m deep water [22]. In the Eastern Tropical Pacific, a genetic study also found two different populations of oceanic manta rays in the Galapagos Islands and island groups off the coast of Ecuador located ~1000 km apart [83]. Based on these recent findings, a detailed genetic study in the Raja Ampat archipelago seems warranted and would provide further insights into population structure and the utility of the metapopulation concept in managing Raja Ampat manta rays. Finally, we are planning further satellite telemetry work in Raja Ampat, specifically targeting *M. alfredi* inhabiting the more remote regions of the archipelago. This work will help better understand the home ranges of these subpopulations and also determine if they frequently leave the boundaries of the Raja Ampat MPA network (a potential management concern).

## 5. Conclusion

Our study provides further evidence that *M. alfredi* in the Raja Ampat archipelago is likely to form a metapopulation composed of at least three subpopulations inhabiting the Ayau, Misool and northwestern Raja Ampat regions. Network analysis of an extensive acoustic telemetry dataset throughout the region revealed high fidelity to specific sites (cleaning stations and feeding sites) within each region, as well as connectivity between several regions through repeated individual movements. We revealed key aggregation sites that are highly connected and influential in the local and regional movements of *M. alfredi*. These sites provide essential services for the long-term viability of this philopatric species. Our study also highlighted the importance of the Eagle Rock cleaning station as a critical node in the *M. alfredi* movement network; the fact that this site, with the second highest degree centrality metric of all sites in Raja Ampat, is currently unprotected and situated outside of Raja Ampat MPA boundaries, is of particular concern. We therefore strongly recommend that this important site is included within the Raja Ampat MPA network. We moreover recommend that the Raja Ampat MPA Management Authority consider refining its approach to the management of the metapopulation of *M. alfredi* in Raja Ampat, creating three management units that each focus on a subpopulation of reef manta rays (Misool, Ayau and northwestern Raja Ampat). Such an approach would encourage more effective management by focusing on specific threats and management concerns in each of these three regions, which have quite different environmental and social settings and different exposures to potential tourism threats.

## Data Availability

Data are provided online [84]. Supplementary material is available online [85].
